# Numerical Simulation of Hemodynamics in Two Models for Total Anomalous Pulmonary Venous Connection Surgery

**DOI:** 10.3389/fphys.2020.00206

**Published:** 2020-03-10

**Authors:** Yeyang Cheng, Aike Qiao, Yao Yang, Xiangming Fan

**Affiliations:** ^1^College of Life Science and Bioengineering, Beijing University of Technology, Beijing, China; ^2^Beijing Anzhen Hospital, Capital Medical University, Beijing, China

**Keywords:** TAPVC, surgical planning, hemodynamics, numerical simulation, congenital heart disease

## Abstract

**Objective:**

To numerically compare the prospective hemodynamic outcomes between a new window surgery and a traditional surgery in the treatment of supracardiac total anomalous pulmonary venous connection (S-TAPVC).

**Methods:**

A 3D geometry model, composed of pulmonary vein (PV) and left atrium (LA), was reconstructed based on summarized data with S-TAPVC. Two surgery models were established based on this model. One is the traditional surgery model, where an elliptical anastomosis was created by incising and stitching the LA and the common vein (CV) along the axis of the CV. The other is the new window surgery model, where the CV was incised with an H-shaped orifice, and LA was incised with a transposed H-shaped orifice, and then the orifice edges were stitched like a window. Two models with a relative cross sectional area (RCSA) of 300 mm^2^/m^2^ and 500 mm^2^/m^2^ were established, which correspond to traditional surgery and window surgery. Numerical simulation of hemodynamics was carried out. The velocity, left atrium and pulmonary vein pressure, the pressure difference of anastomosis and the energy conversion efficiency were analyzed to evaluate the prospective hemodynamic outcomes of these two operations.

**Results:**

Window surgery presented a lower blood flow velocity, pressure difference, and the WSS at the anastomosis, compared to traditional surgery. In terms of energy loss, the power conversion efficiency of window surgery was significantly higher than that of traditional surgery, with 66.8% and 53.5%, respectively.

**Conclusion:**

The new window surgery demonstrates a lower pressure difference of anastomosis and higher energy conversion efficiency, which may be a better choice compared with the traditional surgery for S-TAPVC patient.

## Introduction

**FIGURE 1:**
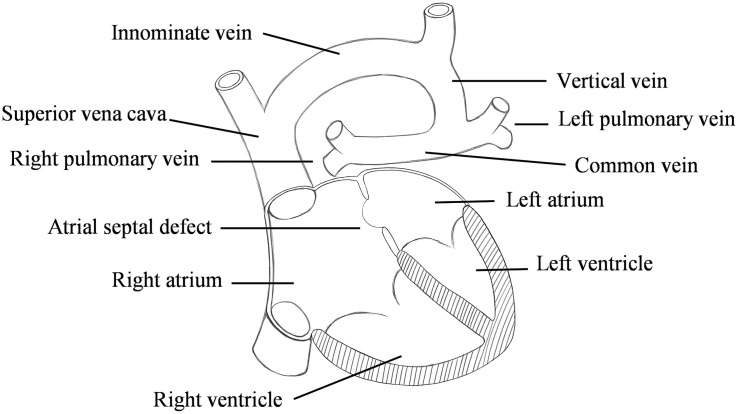
Schematic diagram of the supracardiac total anomalous pulmonary venous connection (S-TAPVC).

Total anomalous pulmonary venous connection (TAPVC) is a rare but serious congenital heart disease (CHD) in which all pulmonary veins (PV) connect to the right atrium (RA) rather than the left atrium (LA) (Hassan et al., 2010; Ho et al., 2018; Figure 1). Its incidence is about 1.5%∼3.6% of CHD (Seale et al., 2010; Zhang et al., 2017; Wu et al., 2019).

Surgical treatment is usually required for this disease, in which anastomosis of the pulmonary vein and left atrium is a key step. The common vein (CV) and LA will be divided parallel along the axis of the former and anastomosed in traditional surgery. However, the therapeutic effect of this operation is poor in physiology and hemodynamics. In addition, pulmonary vein obstruction (PVO) is one of the most frequently stated problems with postoperative death (Seale et al., 2013; Hoashi et al., 2015; Shi et al., 2017).

Recent evidence suggests that the larger anastomotic area of pulmonary vein and left atrium is the key factor to improve the therapeutic effect of TAPVC (Ricci et al., 2003). On the other hand, new surgical procedures have been studied. In the 1990s, sutureless technique to relieve PVO after TAPVC repair, and the early treatment effect was good (Najm et al., 1998; Lacour-Gayet et al., 1999). However, the long-term outcomes of this technique are still unclear (Azakie et al., 2011; Yoshimura et al., 2017; Wu et al., 2019). What’s worse, the surgical procedures cannot completely avoid the occurrence of anastomotic stenosis. In addition, the current research mostly focuses on mathematical statistical analysis of the postoperative mortality and postoperative complications of the disease, and few numerical simulations on the hemodynamics of the disease have been performed. Harada et al. (2018) used the Cox proportional hazard model to study the patient variables that may be associated with time to death. Wu et al. (2019) performed a meta-analysis using the Cochrane *Q* test and the I^2^ statistic to assess heterogeneity to compare the treatment effects of sutureless technique and conventional surgery for primary repair. White et al. (2019) used univariable and multivariable Cox proportional hazard regression methods to identify factors associated with the primary outcome.

Therefore, a new window surgery was proposed in this study, and computational fluid dynamics (CFD) was used to evaluate the prospective hemodynamic effects of the new window surgery.

## Materials and Methods

### Three-Dimensional Model Construction

**FIGURE 2:**
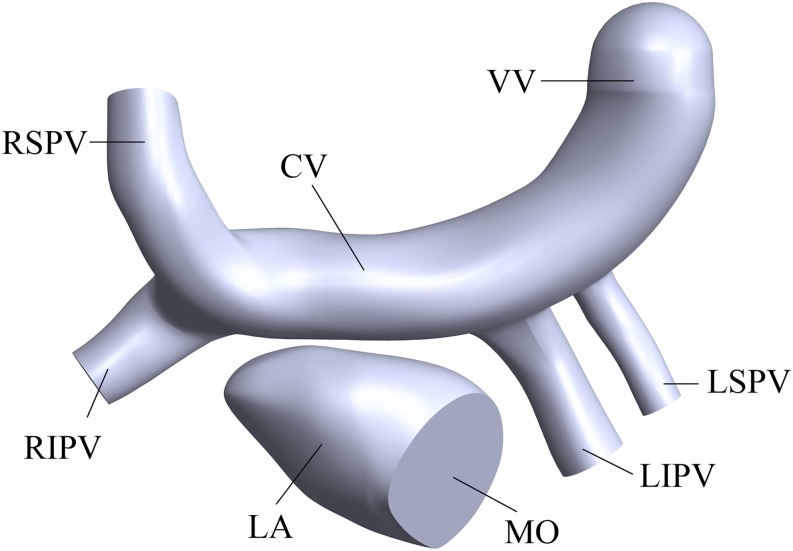
Three-dimensional of the LA and PVs. “LA” means left atrium, “MO” means mitral orifice, “RSPV” means right superior pulmonary vein, “RIPV” means right inferior pulmonary vein, “LSPV” means left superior pulmonary vein, “LIPV” means left inferior pulmonary vein, “CV” means common vein and “VV” means vertical vein.

The simplified three-dimensional (3D) model of supracardiac total anomalous pulmonary venous connection (S-TAPVC) was constructed based on summarized data in the references and the recommendations of the surgeons (Kim et al., 2005; Cronin et al., 2007), including the PV, LA, CV, and part of the vertical vein (Figure 2). It was constructed in the Computer Aided Design (CAD) software of SolidWorks.

### Surgery and Geometry Processing

**FIGURE 3:**
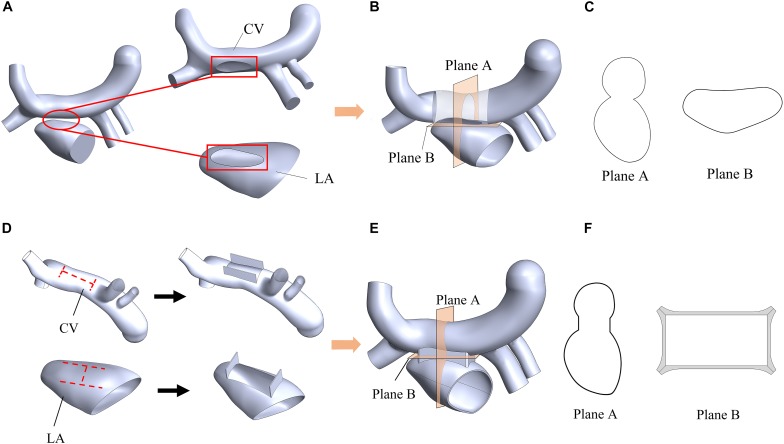
Schematic diagram of a traditional surgery **(A,B,C)** and the window surgery **(D,E,F)**. **(A)** The elliptical anastomoses were created on the CV and LA; **(B)** Two orifice edges were sutured using traditional methods; **(C)** Plane A and Plane B were two sections of the model of the traditional surgery; **(D)** CV and LA were incised with an H-shaped orifice and a transposed H-shaped orifice, respectively; **(E)** These orifice edges faced outward and were sutured; **(F)** Plane A and Plane B were two sections of the model of the window surgery. Plane A was defined as the plane passing through the center of the CV and perpendicular to the CV axis; Plane B was defined as a plane near the anastomosis and perpendicular to Plane A.

In order to evaluate the therapeutic effect of TAPVC, two surgical models were established for comparison. One is the traditional surgery model, where an elliptical anastomosis was created by incising and stitching the LA and the CV along the axis of the CV (Figures 3A,B). The other is the new window surgery model, where the CV was incised with an H-shaped orifice, and LA was incised with a transposed H-shaped orifice, and then the orifice edges were stitched like a window (Figures 3D,E). The cross-sections of the anastomosis of the two models were shown in Figures 3C,F.

The relative cross sectional area (RCSA), which is defined as the ratio of actual anastomotic area to body surface area, is a critical geometric factor with regards to the surgical outcomes. RCSAs for the traditional surgery model and the window surgery model were about 300 mm^2^/m^2^ and 500 mm^2^/m^2^, respectively. RCSAs in the two models have been designed as large as possible.

**FIGURE 4:**
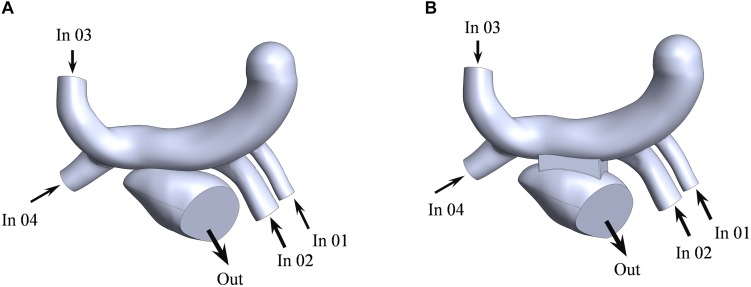
The fluid models of the traditional surgery **(A)** and the window surgery **(B)**. LSPV, LIPV, RSPV, and RIPV were defined as the inlet for numerical simulation, corresponding to In 01, In 02, In 03, and In 04 in the figure, respectively; mitral orifice was defined as the outlet, out corresponding to the figure.

**FIGURE 5:**
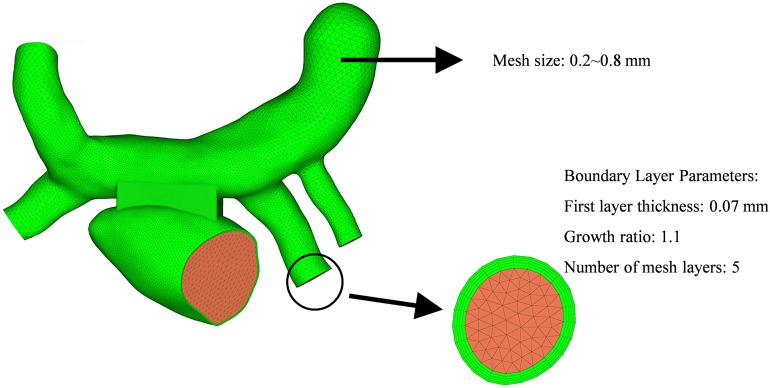
Schematic diagram of the mesh details in a cross section of the vessel.

The computational fluid models were reconstructed in the software of Solidworks for the two surgeries (Figure 4). The mesh was generated by the software of Hypermesh. The mesh density was increased in the region of interest. By performing mesh independency analysis, the feasible mesh size was adopted. The element size on the wall was in the range of 0.2 ∼ 0.8 mm. In addition, the mesh in the boundary layer was created. The parameters of the boundary layer are as follows: the thickness of the first layer is 0.07 mm, the growth ratio is 1.1, and the number of mesh layers is five (Figure 5).

### Materials and Boundary Conductions

The walls of the left atrium and blood vessels were assumed to be rigid and no-slip. The blood was assumed to be an incompressible, Newtonian fluid with density of 1050 kg/m^3^ and viscosity of 0.0035 Pa⋅s (Soulis et al., 2008; Ding et al., 2013; Lantz et al., 2016).

The left atrium is the passage of blood from the pulmonary veins to the left ventricle. The purpose of this study was to explain the effect of window surgery on the function of the left atrium. Therefore, this function can be simulated using steady-state flow. The mass flow of the pulmonary vein was defined as the boundary condition at the inlet (Figure 4). The average flow rate in a cardiac cycle was distributed to four pulmonary veins in proportion to the area of the pulmonary veins (Lantz et al., 2019). In addition, mitral orifice was defined as the outlet boundary condition of computational fluid model with average left atrial pressure (Migliavacca et al., 2003), as shown in Table 1. In clinical surgery, the vertical vein is usually ligated. Therefore, it was neither the inlet nor the outlet in the computational fluid model.

**TABLE 1:** The setting of the boundary conditions.

	Inlet flow rate (kg/s)				Outlet pressure (mmHg)
Location	In 01	In 02	In 03	In 04	Out
Parameter	0.0075	0.0189	0.0214	0.0159	4.0

### Numerical Computation

Based on the abovementioned models and boundary conditions, the flow field was computed by using CFX 14.5 (Ansys, United States) on a computer with Intel Core i5-4590 processors at 3.30 GHz and 8 GB RAM. Analysis type was set to steady state. In solver control panel, advection scheme was set to high resolution, and the maximum iteration steps were set to 200. Furthermore, the residual tolerance was set to 1.0e-4 in order to make the calculated result within an acceptable range.

The Reynolds number (Re) was defined as

(1) $$\text{Re}=\frac{\rho \,v\,d}{\mu }$$

where the ρ, v, d, μ are the density, velocity, the hydraulic diameter and the dynamic viscosity of blood, respectively (Table 2).

**TABLE 2:** The velocity, diameter, and Reynolds number of blood in the four pulmonary veins.

	LSPV	LIPV	RSPV	RIPV
Velocity (m/s)	0.476	0.474	0.485	0.473
Diameter (m)	0.0044	0.0069	0.0073	0.0064
Density (kg/m^3^)	1050	1050	1050	1050
Dynamic viscosity (Pa⋅s)	0.0035	0.0035	0.0035	0.0035
Reynolds number	629	981	1063	909

Considering that the maximum Reynolds number (Re) was approximately 1063, the blood flow was assumed laminar.

### Energy Loss

Energy loss is an important indicator for evaluating hemodynamic performance. This study evaluated the energy loss by the power loss and the power conversion efficiency (Guadagni et al., 2001; Migliavacca et al., 2003).

The power loss was defined as

(2) $$\text{W}=\text{Q}\,\left(\text{P}+\frac{1}{2}\,\rho \,v^{2}\right)$$

(3) $${\text{W}}_{l}=\sum W_{i\,n}-\sum W_{o\,u\,t}$$

where the W, Q, P, ρ, v are the power, mass flow, static pressure, the blood density and the blood velocity, respectively. The W_*l*_ means the power loss, and ∑*W*_*in*_∑*W*_*out*_ are the total power of inlet and outlet, respectively. What’s more, the power conversion efficiency (e) was defined as:

(4) $$e=1-\frac{W_{l}}{\sum W_{i\,n}}$$

## Results

### Flow Patterns

**FIGURE 6:**
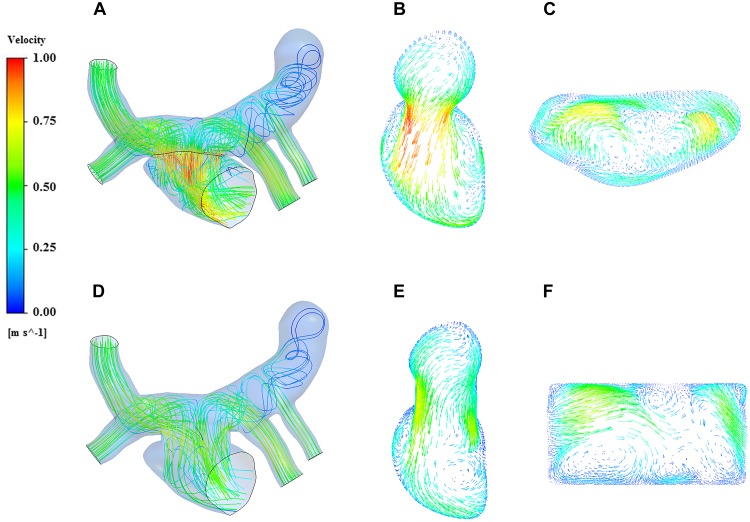
Streamline and velocity vector diagrams of the two models. **(A)** streamline of traditional surgery; **(B)** velocity vector on the Plane A of traditional surgery; **(C)** velocity vector on the Plane B of traditional surgery; **(D)** streamline of window surgery; **(E)** velocity vector on the Plane A of window surgery; **(F)** velocity vector on the Plane B of window surgery.

The streamline reflects the magnitude and direction of the flow velocity at each point in the fluid domain. The streamlines of the two models could be observed clearly in the Figure 6, and the region with high blood flow velocity was located near the PV-LA anastomosis in both of the models. The blood flow velocity of traditional surgery was significantly higher than that of the window surgery with 1.0 m/s and 0.8 m/s, respectively.

On the other hand, blood in the pulmonary veins flowed into the left atrium smoothly in the window surgery model. A complex vortex extended from the anastomosis to the bottom of the left atrium along the left atrial wall in the traditional surgery model, which seemed not obvious in the window surgery model. There were low-velocity blood flows in the vertical vein on both models, and the vortices produced by traditional surgery were more obvious.

### Pressure Distribution

**FIGURE 7:**
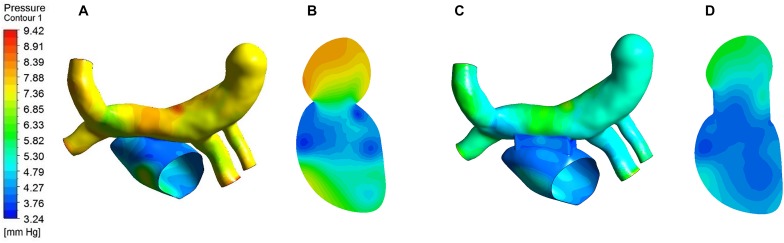
Pressure distribution of the two models. **(A)** Pressure distribution on the wall of traditional surgery; **(B)** Pressure distribution of Plane A in traditional surgery; **(C)** Pressure distribution on the wall of window surgery; **(D)** Pressure distribution of Plane A in window surgery.

The pressure distribution contour of the two models obtained from the analysis of CFD was showed in Figure 7. On the whole, the pressure of traditional surgery on the wall was higher than that of window surgery. The high-pressure region of traditional surgery focused on the CV and pulmonary veins, with the value up to 9.42 mmHg, while in window surgery, the pressure distribution was relatively uniform. In terms of pressure at the PV-LA anastomosis, the pressure difference was defined as the difference between the average pressure in the CV region and the average pressure in the left atrium region on the plane A, which was a section of the models. The pressure difference of conventional surgery was greater than that of window surgery, with approximately 3 mmHg and 1 mmHg, respectively.

### Wall Shear Stress

**FIGURE 8:**
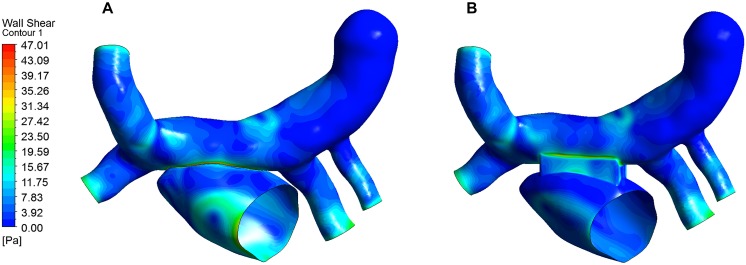
WSS distribution of the two models. **(A)** WSS for the traditional surgery; **(B)** WSS for the window surgery.

Wall shear stress (WSS) is an important parameter in hemodynamic analysis, and many studies have shown that WSS was associated with remodeling of blood vessels (Farmakis et al., 2004; Fukumoto et al., 2008; Pekkan et al., 2009). The WSS distribution was extracted and shown in Figure 8. The highest WSS in both models were located near the anastomosis, and that of the traditional surgery was larger than that of the window surgery, with 47.01 Pa and 29.47 Pa, respectively.

### Power Loss

Power loss can visually reflect the work of blood flow, and has a positive effect on evaluating the expected therapeutic effects of different surgical procedures on TAPVC. The results of the power loss and the power conversion efficiency of two models can be calculated and compared in Table 3. There is a significant reduction in power loss from 32.8 mW in the traditional surgery to 17.7 mW in window surgery. In terms of the power conversion efficiency, the window surgery has increased from 53.5 to 66.8% compared with the traditional surgery.

**TABLE 3:** Power loss and power conversion efficiency.

	Traditional surgery	Window surgery
Power loss (mW)	32.8	17.7
Power conversion efficiency (%)	53.5	66.8

## Discussion

The left atrium is the channel through which blood in the pulmonary veins flows back to the left ventricle. A smooth blood flow state from the pulmonary vein to the left atrium is an important condition for ensuring left ventricular function. Local disturbed flow may cause anastomotic stenosis (Honjo et al., 2010; Azakie et al., 2011; Yoshimura et al., 2017). In clinic, it is generally considered that anastomotic stenosis and PVO are prone to occur when the blood flow velocity at the PV-LA anastomosis is greater than 1.2 m/s. From the results of numerical simulation, the blood flow velocity at the anastomosis of the two surgical procedures was less than 1.2 m/s, and the window surgery demonstrates lower velocity than the traditional surgery. We believe this is the result of the window surgery which increased the PV-LA anastomotic area.

In clinical treatment, high pressures in the pulmonary vein and the left atrium aren’t what the surgeon expects. In the present study, the maximum pressure region of the two models was located on the opposite side of the junction of LIPV and the CV due to the impact of blood flow from the LIPV on the CV. On the other hand, it is interesting to note that the pressure and the pressure differences between the pulmonary veins and the left atrium of window surgery were lower than those of the traditional surgery. This is because the former is able to provide a larger area of the PV-LA anastomosis, which is beneficial to the reduction of the resistance of the lungs to the left heart system.

The heart pumps blood all the time to ensure the normal life of the body. If the heart has too much ineffective work, it will not only affect the normal physiological function of the human body, but also increase the load on the heart, which is unfavorable to the health of the patient from the perspective of long-term outcomes. In order to analyze long-term efficacy, power loss was calculated. In the window surgery, the structural transition from the pulmonary vein to the left atrium is more gradual, avoiding the structure of sudden expansion and contraction in traditional surgery, and blood in the pulmonary vein flows more smoothly into the left atrium and energy loss can be reduced. The results of numerical simulation confirmed that the window surgery has a positive effect of on the treatment of TAPVC in terms of reducing power loss and increasing power conversion efficiency.

Previous studies have noted that WSS is closely related to the function and structure of endothelial cells, and suggested that when WSS > 35 Pa or WSS < 0.2 Pa, intimal hyperplasia has the possibility of proliferation (Fry, 1968; Meyerson et al., 2001; Cunnane et al., 2017; de Villiers et al., 2018). The WSS of this study were all greater than 0.2 Pa, and WSS in the window surgery was less than 35 Pa, and WSS in the traditional surgery was greater than 35 Pa. It may be related to the structure of the anastomosis in the traditional surgery and the complex changes in blood flow velocity at this location. So we speculate that traditional surgery may increase the possibility of intimal hyperplasia. But this view needs further verification.

## Limitations

The reconstructed CFD model and boundary conditions were simplified. In the future work, more patient-specific models should be established and their boundary conditions should be obtained to ensure the accuracy and validity of the numerical simulation results. In addition, the pulmonary veins and the left atrium were assumed to be rigid walls, and the interaction between blood flow and the wall was not considered, which may have an impact on the calculated results. Fluid-solid coupling methods should be considered to simulate the treatment of TAPVC in different surgical procedures in future work. Finally, it would be better to confirm the conclusion in animal experiments.

## Conclusion

This preliminary study demonstrates that the proposed window surgery has some advantages over the traditional surgery in the treatment of TAPVC. The window surgery presented a good hemodynamic performance, which allowed smoother flow of blood from the pulmonary veins into the left atrium, reduced the pressure difference between the pulmonary veins and left atrium, and increased the power conversion efficiency. In summary, the window surgery may provide a new surgical alteration for the treatment of TAPVC and provide a reference for clinicians to develop new surgical planning.

## Data Availability Statement

All of the data produced and analyzed in the present study are involved in the manuscript as tables or figures. The corresponding author will respond to the requests concerning the raw data and reasonable accommodations will be provided.

## Author Contributions

YC performed numerical simulation of computational fluid dynamics and performed the data analysis under the instruction of AQ. YY and XF provided the support of medical knowledge. The initial manuscript draft was prepared by YC and subsequently revised by AQ. All authors approved the final submitted version.

## Conflict of Interest

The authors declare that the research was conducted in the absence of any commercial or financial relationships that could be construed as a potential conflict of interest.
